# Modulatory role of garlic ( *Allium sativum*) extract against cisplatin- induced nephrotoxicity in female albino rats and their offspring

**DOI:** 10.12688/f1000research.111293.1

**Published:** 2022-05-09

**Authors:** Abdelfattah Elbeltagy, Gamal Mohamed, Mohammed Akeel, Karoline Abdelaziz, Kadry Elbakry, Ahmed Elsayed

**Affiliations:** 1 Zoology, Damanhour University Faculty of Science, Damanhour, 22511, Egypt; 2 Department of Human Anatomy, , Faculty of Medicine, Jazan University, Jazan, KSA, Jazan, 45142, Saudi Arabia; 3 Zoology, Faculty of Science, Damietta, University, Damietta, Egypt, Damietta, 34611, Egypt

**Keywords:** Cisplatin, nephrotoxicity, histopathology, garlic, antioxidants, caspase3

## Abstract

**Background**: Cisplatin (CP) is one of the chemotherapeutic drugs widely utilized in the treatment of several malignancies. However, recently; its use has been limited because of its hazardous health drawbacks. Previous researches confirmed that CP has severe deleterious side effects on pregnant mothers and their fetuses. Garlic ( *Allium sativum*) extract has been claimed to exhibit potent antioxidative and free radical scavenging abilities.

**Aim:** This work is mainly designed to evaluate the potential therapeutic role of garlic extract against CP-induced nephrotoxicity in pregnant rats and their offspring.

**Methods:** 24 pregnant rats were used in the current study. They were randomly allocated into four groups (n=6):  control, garlic, CP, and CP + garlic group. At the end of the weaning period, the mothers and the offsprings of all groups were sacrificed, the kidneys were immediately excised, and processed for histological and biochemical investigations. Also, blood samples were withdrawn and processed for estimation of the assigned biochemical parameters.

**Results:** The renal histological sections from CP-treated mother rats displayed pronounced histopathological lesions however, their offspring showed mild renal histopathological lesions if compared with those of their mothers. The levels of renal tissue Superoxide dismutase, catalase, and glutathione peroxidase enzymes were significantly decreased. On the contrary, the levels of malondialdehyde, serum urea, and creatinine were significantly increased in CP-treated mother rats and their offspring as compared with control. The percentage value of caspase 3 activity was markedly elevated in the renal tissues of CP-treated mother rats and their offspring compared to the control group. Supplementation of garlic extract to the CP treated rats; the overall histological lesions, as well as biochemical parameters, were restored nearly to the control ones. It is concluded that garlic ( *Allium sativum*) extract has a powerful ameliorative role against CP-induced nephrotoxicity in pregnant rats and their offspring.

## Introduction

Cisplatin (CP), carboplatin, and oxaliplatin are platinum-based chemotherapeutic drugs. ^1^ These drugs persist for a long time in the blood and bind to serum proteins, especially to the serum albumin. ^2^ Generally, all these platinum drugs can induce nephrotoxicity through acute tubular necrosis of proximal tubular cells, ^3^^,^^4^ but it is most commonly associated with CP. ^5^

CP is mostly used for treatment of solid cancers, like bladder cancer, ^6^ head and neck cancers, ^7^ cervical cancer, ^8^ testicular cancer, ^9^ ovarian cancer, ^10^ and pulmonary cancers. ^11^ Experimental studies on rats and mice revealed that CP leads to nephrotoxicity through formation of glutathione conjugates in blood stream. ^12^ Other studies showed that CP binds to DNA leading to arrest of DNA synthesis and replication resulting in cell apoptosis. ^13^^,^^14^ Moreover, Karasawa *et al*. stressed that **,** the nephrotoxicity induced by CP is mainly attributable to toxic generation of free radicals and binding to necessary cytoplasmic molecules, especially the anti-oxidant glutathione. ^15^ CP also suppresses antioxidant enzymes like superoxide dismutase (SOD). ^16^ Furthermore, the nephrotoxicity induced by CP is associated with elevated serum creatinine and urea, in addition, traces of glucose and protein may appear in urine. ^17^ A specific study on humans revealed that CP has a high rate of placental transfer whereas the concentration of CP in the maternal blood is approximately equal to that found in the umbilical cord blood. ^18^

Natural products from plants and animals have been widely utilized all over the world either in the pure forms or crude extracts for protection from or treatment of various diseases. ^19^ *Allium sativum* (Garlic) is one of the most famous plants that is widely used to combat several diseases because it contains more than 33 sulfur compounds, enzymes, minerals, amino acids, vitamins including A, B1 and C as well as fibers. ^20^ The most vital active sulfur compounds in *Allium sativum* are allicin and alliin which are considered the main antioxidants and scavenging free radical compounds. ^21^ Zaidi *et al*. declared, allicin and alliin can improve the hepatic functions via regulation of circulating liver enzymes. ^22^ Also, garlic extract has a powerful role in lowering the levels of blood cholesterol, low density lipoprotein (LDL), triglycerides, ^23^ urea as well as blood glucose levels in diabetic mice and rabbits. ^24^

Garlic extract is used to lessen abdominal discomfort, diarrhea and infections of respiratory tract, ^25^ and for antimicrobial action. ^26^ It can help to restrain some forms of cancers, heart disease, strokes and viral infections, ^27^ coronary diseases, ^28^ common cold, ^29^ and prevention of neurotoxicity. ^30^ Previous studies confirmed that CP can penetrate the placental barrier and induce congenital malformation in fetuses. ^31^ On the other hand, placental transfer of garlic components was recorded by Saleh *et al*. ^32^

Consequently, the current investigation was designed to evaluate the therapeutic role of garlic extract on the nephrotoxicity induced by CP in pregnant female rats and their offsprings.

## Methods

### Ethical approval

The ARRIVE guidelines were followed, ^91^ and a completed checklist is available at. All efforts were made to ameliorate the suffering of animals by keeping the animals inside clean conditions provided with suitable humidity, regular dark light cycle and gentle injection of cisplatin dose. This study was approved by the Bioethics Committee of Damanhur University, no. EA 23122, 2020. All experiments inclusive of animal handling and sacrifice were conducted as per the guidelines of the Bioethics Committee of Damanhur University.

### Chemical

CP was obtained in from of commercial Egyptian Unistin Vial from Egyptian International Medical Company.

### Preparation of garlic extract

One kg of fresh garlic was obtained from the local market in Damanhur city, peeled and crushed by a blender and filtered through a strainer to obtain aqueous suspension. The obtained suspension was diluted in distilled water at 4 g/mL then centrifugated (Grant Instruments, LMC-3000 laboratory centrifuge, Cat.No. BIOSAN_22005) at 3000 ×g for ten min to separate the hard fibers.

### Experimental animals

In the current investigation, 24 females and 8 males of adult Wistar rats were obtained from the Hellwan Breeding Farm, Ministry of Health, Cairo, Egypt. The rats were housed in plastic cages in a well ventalated animal house under highly sterilized conditions at 25±2°C, appropriate humidity (50±10%) and regular cycle of 12 h light/12 h dark. They were fed on standard food and water *ad libitum.* After two weeks of acclimatization, the rats were mated in 8 special cages (1 male:3 females). After confirmation of pregnancy by investigation of vaginal smear for each female rat, the pregnant rats were isolated and randomly categorized into 4 groups (n=6) as follows:

**Group I** (control): The pregnant rats received 0.5 ml of distilled water daily through the oral route by a modified plastic syringe (intra-gastric tube).

**Group II** (Garlic extract): The pregnant rats received a daily oral dose of garlic extract (250 mg/kg) from the day-4 of gestation till the end of weaning. ^33^

**Group III** (Cisplatin): The pregnant rats received a single intraperitoneal dose of CP (5 mg/kg b.w) at the 4 ^th^ day of pregnancy, followed by 0.5 ml of distilled water daily through the oral route till the end of weaning. ^34^

**Group IV** (CP and Garlic extract): They received a single I. P dose of CP as in group III followed by a daily oral dose of garlic extract (250 mg/kg b.w) till the end of weaning.

### Sample collection and tissue preparation

At the end of weaning (21 ^st^ day post-natal), the mother rats and their 21 day old offspring were weighed and sacrificed by decapitation. The blood samples were immediately withdrawn by direct cardiac puncture and the serum was separated by centrifugation (Laboratory Centrifuges, R-8C) at 3000 rpm and stored frozen at -20 ^o^C for estimation of biochemical parameters. ^88^ The rats were dissected through a midline abdominal incision and the kidneys were immediately excised and washed in normal saline. The right kidneys were longitudinally cut into two equal halves and kept in 10% neutral formalin for histological study. On the other hand, the left kidneys were homogenized using a Potter-Elvenhjem homogenizer in ice cold 1.15% potassium chloride (3mL per 1g of tissue) followed by centrifugation at 5000 rpm for 15 min and of the supernatant was separate and kept frozen for evaluation of antioxidants.

**Investigated parameters**

***Histological examination of kidneys***

The right kidneys from mother rats and their 21 day old offspring were immediately fixed in 10% formalin for two days, dehydrated in ascending series of ethanol, cleared in xylol and put in melted paraffin wax. After cooling, the paraffin blocks were held in a Leica RM microtome (Minux ^®^, S700 Rotary Microtome). 4-5 μm serial sections of renal tissues were cut and put on clean glass slides. The obtained sections were immersed in descending grades of alcohol and put in the oven to remove the wax using xylene. Finally, the sections were stained by hematoxylin and eosin (H&E) for investigation the histological architecture of kidneys using an Olympus BH-2 light, Cat. No. 6924 BH,) microscope fitted with digital camera (Canon EOS 6d mark II) to take photomicrographs.

***Serum analysis***

*Assessment of creatinine and urea*

Serum creatinine and urea were estimated using criterion laboratory protocol and applying the commercially obtainable earmark kits by an auto analyzer (BT 300, Japan,Cat.No.NX-600-E210). ^35^

*Assessment of sodium, potassium and magnesium*

The levels of serum sodium and potassium were measured using Ireland E2A Na ^+^/K ^+^ reagent kits. Serum magnesium was determined by a spectrophotometer system using kits from BioMeriecux- France,Cat.No. 031 685858.

***Assessment of antioxidants and MDA in renal tissues***

*Measurment of MDA and antioxidant enzymes*

A part of the aliquots of the supernatant of left renal tissue homogenate was used to measure the levels of superoxide dismutase (SOD), catalase (CAT), Glutathione peroxidase (GPx) and malondialdehyde (MDA). The levels of MDA were assayed by measuring the level of thiobarbituric acid reactive substances (TBARS) that was used to evaluate the liberated MDA as a result of lipid peroxidation of cell membranes. ^36^

The levels of SOD, CAT and GPx were detected by a colorimetric protocol using trade kits (Biodiagnostic, Cairo, Egypt, Cat No: E-BC-F006). The levels of SOD in the renal tissues homogenate were measured on basis of the ability of the SOD to supress the reduction of nitroblue tetrazolium (NBT). ^37^ The levels of CAT enzyme were measured according to protocol evaluated by Aebi. ^38^ GPx was detected in the kidney homogenates using commercial kits (Biodiagnostic, Cairo, Egypt, Cat.No. MBS744364) based on the industrial ^,^s orders. ^39^

***Flow cytometric detection of Caspase-3***

The activity of caspase-3 was detected by flow cytometry technique in order to inspect the number of apoptotic cells in the renal tissues for groups 1, 3&4. This method is viable where the fluorochrome is immediately binded to the first antibody (Phycoerythrin (PE) and Fluorescein isothiocyanate (FITC) conjugate). The renal cell suspension was set to a density of 1 × 10 ^6^ cell/ml with PBS/BSA buffer (phosphate buffered saline and 1% bovine serum albumin). Aliquot of 100μ L of renal cell suspension was placed test tubes as needed. The antibody (FITC rabbit anti-active caspase-3, solid as, material No.559341, catalog No. 554714, from BD Pharmingen) was added to 10μ L for each sample, mixed fully, and incubated at 25°C for 30 min. thereafter the cells were washed in 2 ml of PBS/BSA followed by centrifugation at 1500 rpm for 5 min and supernatant was removed. The cells were re-suspended in with 0.2 ml of 0.5% paraformaldehyde in PBS/BSA. The data was obtained by flow cytometry. ^89^ This protocol was done in the Mansoura University Hospital using FACS Calibur Flow Cytometer (Becton Dickin-son, Catalog-4. A1 - Anesthesia Products Sunnyvale, CA, USA) equipped with a compact air-cooled low power 150 mW multi-line,4lines selecatble Argon ion laser beam, Cat. No. 58-472 (488 nm).

### Statistical analysis

Data were expressed as mean ± standard error, (n=6 per group). Statistical analysis: one-way analysis of variance (ANOVA) followed by post hoc test. All statistical analysis was completed using the Analysis ToolPak in Microsoft Excel (Microsoft, Excel 2019) (RRID:SCR_016137). Means in the same row with different superscript (*) are significantly different (p > 0.05), *significant at p-value > 0.05, ** significant at p-value > 0.01 and ***significant at p-value > 0.001.

## Results

### Body weight changes

**Figure 1.**
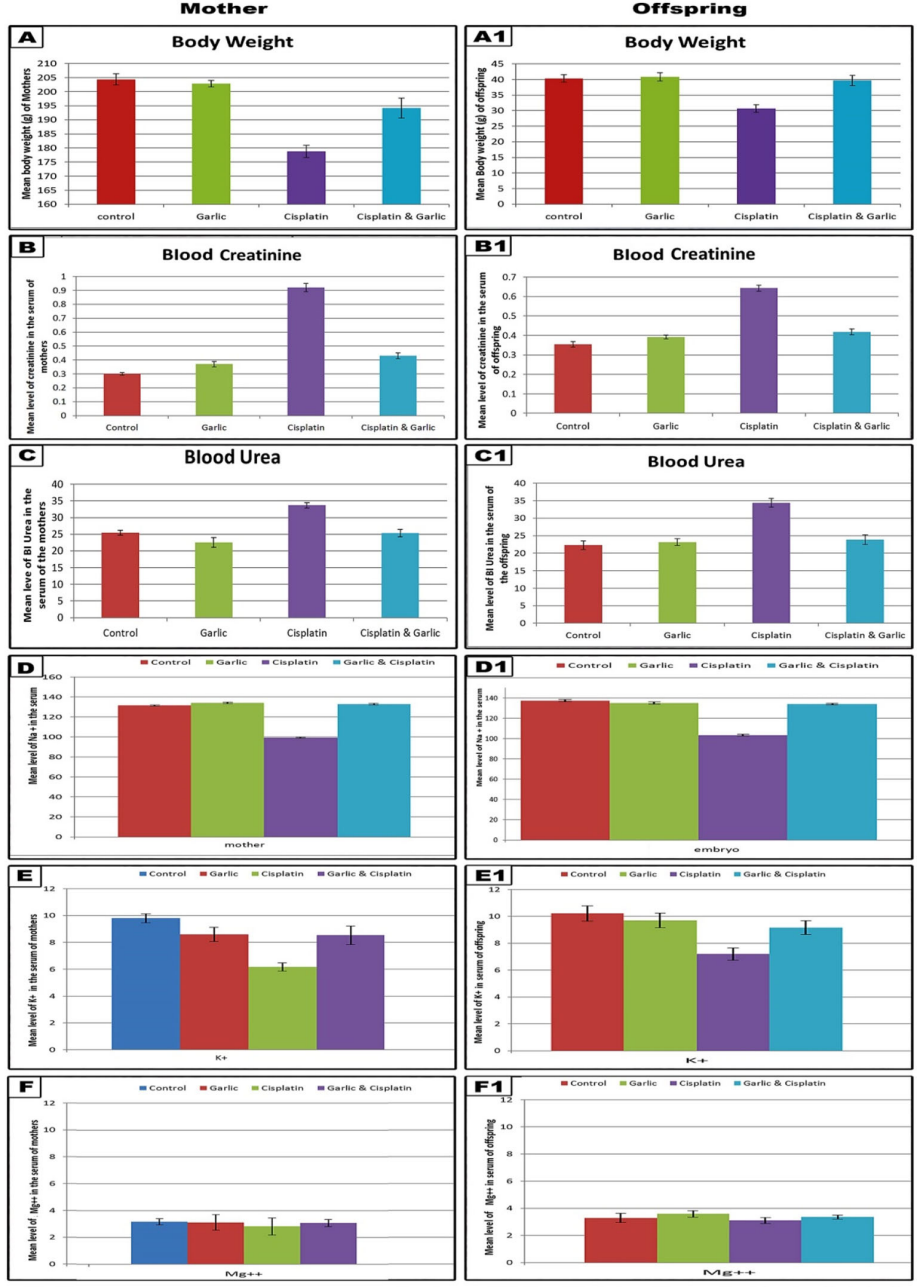
A graphical histogram illustrating the body weight changes, the levels of blood creatinine and electrolytes (Na, K and Mg) among all studied groups of mother's rats and their pups. Note, a highly significant decrease in the mean body weight, blood sodium & potassium and a significant increase in the blood creatinine and urea among cisplatin induced in mother rats and their offspring. Following administration of cisplatin and garlic extract, the levels of blood creatinine in mother rats and their pups was still showing a significant increase (low significant and moderate significant respectively) with control while the level of urea and electrolytes are restored to the normal value as control. (The software used to edit the images and labels: Adobe photoshop CS 8me (Adobe, US) (RRID:SCR_014199) similar results can be achieved using Microsoft Paint).

The obtained results revealed that, the mean body weights of CP injected mother rats and their offsprings were significantly lowered (p < 0.001) if compared with control. Following concomitant administration of CP and garlic extract, the mean body weight was markedly elevated (p < 0.001) if compared with CP group but still showing, a low significance, decrease for mothers and non-significant change for their offsprings if compared with the control values ( Table 1 and Figure 1 A&A1). ^90^

**Table 1.** The mean levels of body weight (g), serum creatinine (mg/dl), urea (mg/dl) and electrolytes (Na, K&Mg) of the mother rats and their offspring.

Parameter		Groups			
		Control n=6	Garlic n=6	Cisplatin (CP) n=6	CP & Garlic n=6
B.wt	Mothers	204.33 ± 1.99	202.83 ± 1.14	178.83 ± 2.15 ^a(^ *** ^)^	194.17 ± 3.55 ^a(^ * ^),b(^ *** ^)^
	21 day-offsprings	40.33 ± 1.2	40.83 ± 1.35	30.67 ± 1.2 ^a(^ *** ^)^	38.67 ± 1.6
Creatinine (mg/dl)	Mothers	0.31 ± 0.01	0.37 ± 0.018	0.92 ± 0.03 ^a(^ *** ^)^	0.43 ± 0.02 ^a(^ * ^),b(^ *** ^)^
	21 day-offsprings	0.35 ± 0.014	0.39 ± 0.01	0.64 ± 0.015 ^a(^ *** ^)^	0.42 ± 0.015 ^a(^ ** ^),b(^ *** ^)^
Urea (mg/dl)	Mothers	25.46 ± 0.67	22.5 ± 1.45	33.67 ± 0.78 ^a(^ *** ^)^	25.35 ± 1.06
	21 day-offsprings	22.27 ± 1.4	23.17 ± 0.98	34.4 ± 1.26 ^a(^ *** ^)^	23.85 ± 1.35
Sodium (mEq/L)	Mothers	131.65 ± 0.71	134.05 ± 0.71	99.28 ± 0.45 ^a(^ *** ^)^	132.88 ± 0.78
	21 day-offsprings	137.35 ± 1.1	135.13 ± 1.1	103.42 ± 0.91 ^a(^ *** ^)^	134.15 ± 0.74
Potassium (mEq/L)	Mothers	9.8 ± 0.33	8.6 ± 0.53	6.17 ± 0.3 ^a(^ *** ^)^	8.53 ± 0.69
	21 day-offsprings	10.22 ± 0.57	9.7 ± 0.55	7.2 ± 0.45 ^a(^ *** ^)^	9.17 ± 0.52
Magnisium (mEq/L)	Mothers	3.17 ± 0.23	3.12 ± 0.57	2.8167 ± 0.64	3.07 ± 0.27
	21 day-offsprings	3.28 ± 0.34	3.58 ± 0.24	3.1 ± 0.22	3.35 ± 0.15

Data are expressed as means ± Standard error, (n=6 per group). Statistical analysis: one-way analysis of variance (ANOVA) followed by post hoc test. Means in the same row with different superscript letters are significantly different (p < 0.05). When p < 0.05,

* Significant at p-value ≤ 0.05,

** Significant at p-value ≤ 0.01 and

*** Significant at p-value ≤ 0.001.

### Changes in serum creatinine and urea

In CP-treated mothers and their pups, the levels of serum creatinine and urea appeared significantly higher (p < 0.001) than control and garlic supplemented groups. Following administration of garlic extract to CP-treated group, the level of serum creatinine appeared significantly lowered (p < 0.001) in comparison with CP alone but still showing, a low significance increase for mothers and a moderately significant increase (p < 0.01) for their offspring if compared with the control group. On the other hand, the level of serum urea, more or less, simulated the values of the control group for both mothers and their offspring in group 4 ( Table 1 and Figure 1 B-C1).

### Changes in serum electrolytes

The results of our study revealed that the levels of serum sodium and potassium ions were significantly decreased (p < 0.001) in CP-treated mothers and their offspring while the levels of serum magnesium exihibited non-significant variation (p > 0.001) if compared with the control group. Following administration of CP and garlic extract, the levels of serum sodium and potassium were improved and showed non-significant change (p > 0.001) in comparison with the control ( Table 1 and Figure 1 D-F1).

### Histological findings

**Figure 2.**
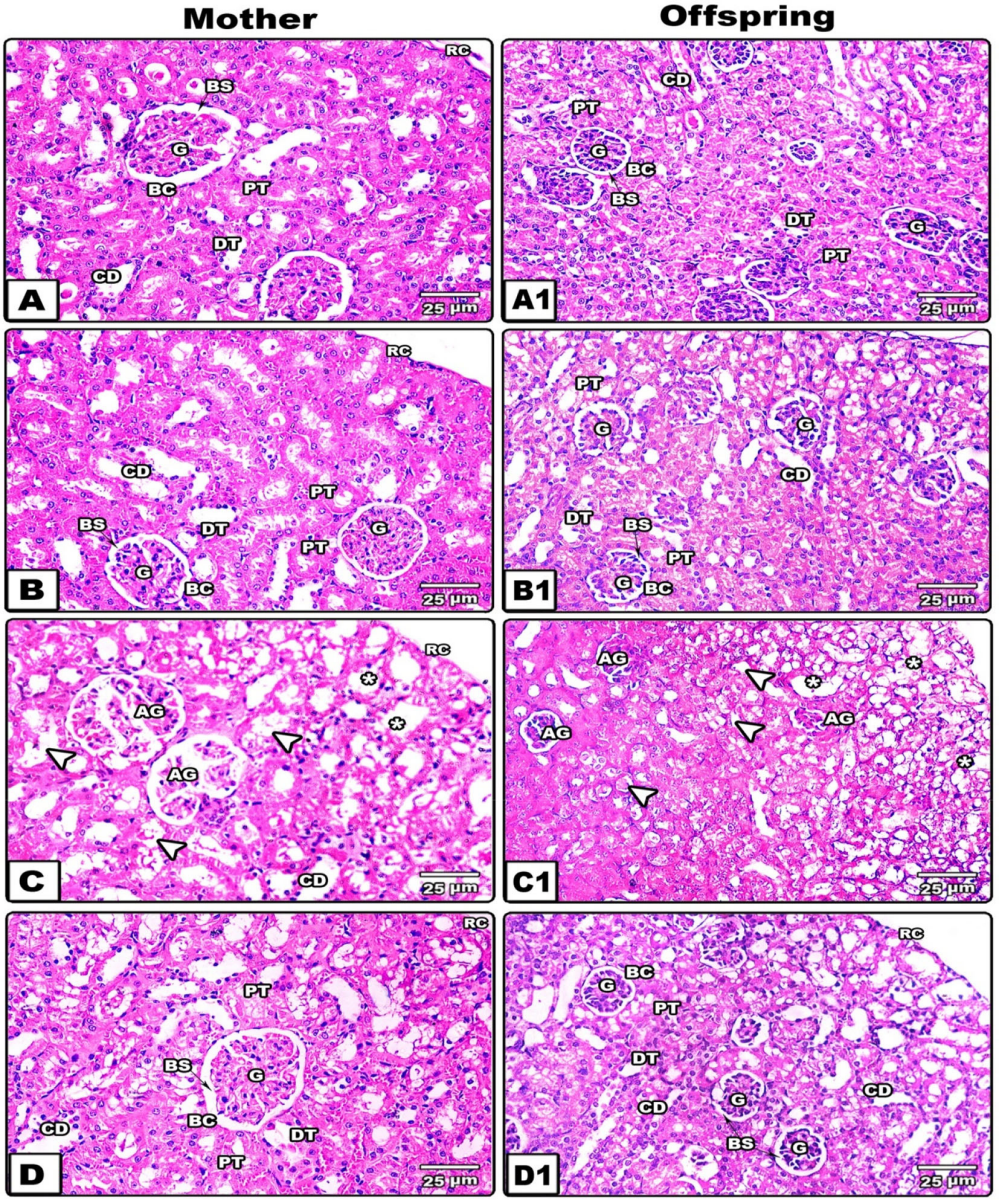
Photomicrographs of histological sections through the renal cortex of the different studied groups of mother's rats and their pups. (A & A1: control, B & B1: garlic, C & C1 cisplatin and D & D1 Following concomitant administration of cisplatin and garlic extract.) Note: the renal cortex appears with normal histological structure of renal corpuscles and tubules in images A-B1, little and atrophied glomerulus (AG), damaged (arrowhead) and dilated tubules (stars) in image C & C1 and obvious amelioration in the histological structure of renal cortex in images D&D1. *(The software used to edit the images and labels: Adobe photoshopCS8me* similar results can be achieved using Microsoft Paint *).* Abbreviations: AG; Atrophied glomeruli, BC; Bowman's capsule, BS; Bowman's space, CD; Collecting duct, DT; Distal tubules, G; Glomerulus, PT; Proximal tubules **,** RC;Renal capsule **×** 400 (H&E).

In control and garlic treated mother rats ( Figure 2 A&B) and their offsprings ( Figure 2 A1&B1) the renal cortex appeared with normal histological structure of renal tubules and renal corpuscles. The renal corpuscle consists of glomerulus (tufts of blood capillaries) that lies in Bowman’s space and surrounded by a tight Bowman’s capsule. The renal tubules are made of well-organized proximal tubules (PT), distal tubules (DT) and collecting ducts (CD). The PT has star-shaped lumen that lined with brush bordered endothelium. The DT has relatively rounded lumen that are surrounded by cubical epithelium with little microvilli. The CD has a relatively wide lumen compared to the PT and DT which is lined with short cubical epithelium.

In CP treated mother rats ( Figure 2 C) and their offsprings ( Figure 2 C1), the renal cortical sections dispalyed remarkable deleterious histological alterations but the severity of alteration was pronounced in mother rats. Such alterations included low density of renal corpuscles with a relativelly wide Bowman’s space, atrophied glomeruli, degenerative tubular cells and cortical tubular dilation. Following administration of CP and garlic extract, the renal cortical sections displayed remarkable amelioration in their architecture in spite of little degenerative tubules being present in some area of the renal cortical section of mothers ( Figure 2 D&D1).

### Changes in renal tissues antioxidants and MDA

**Figure 3.**
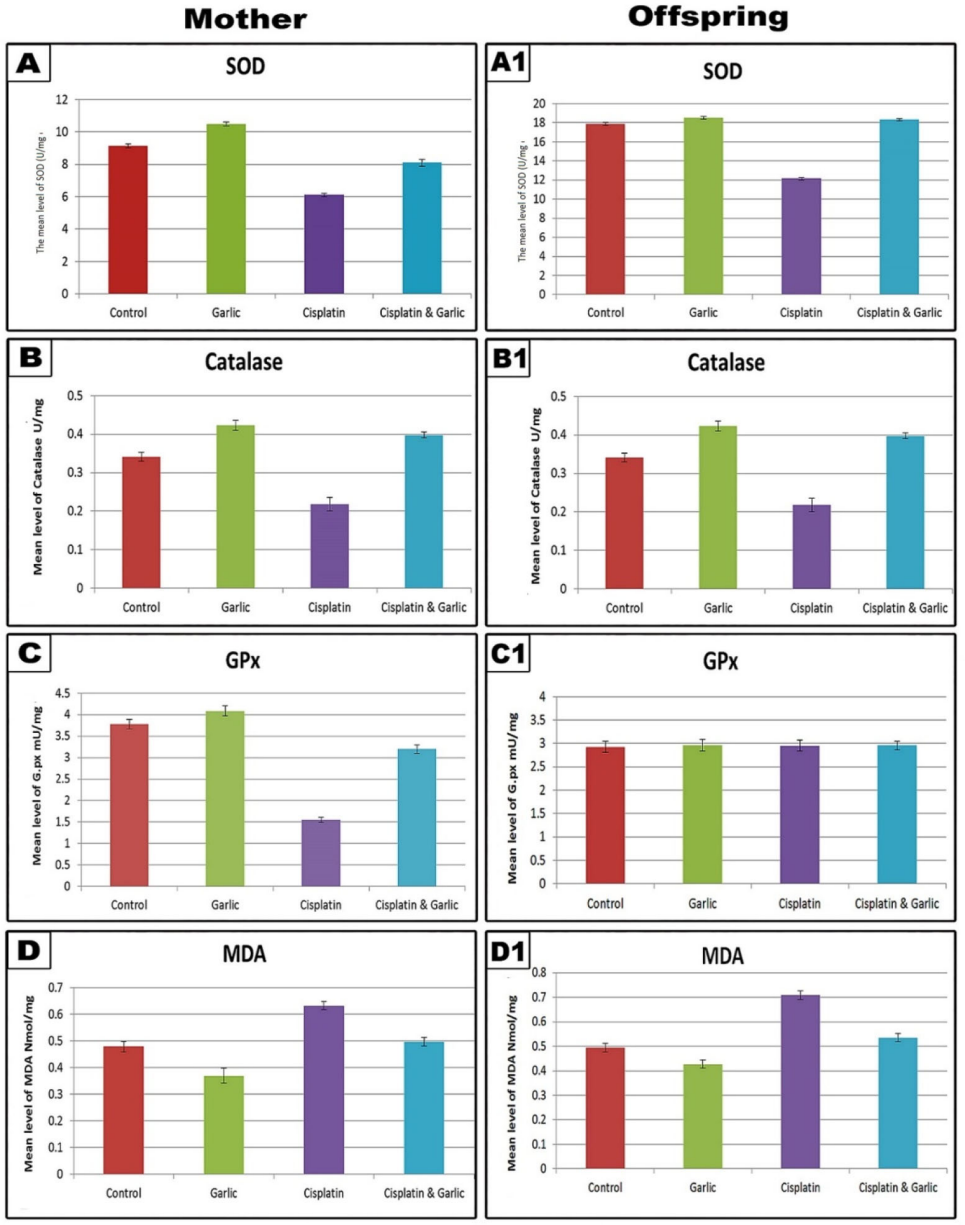
Graphical histogram illustrating the levels of antioxidants (Superoxide dismutase (SOD), catalase and glutathione peroxidase (GPx), and malondialdehyde (MDA) in the renal tissue of the different studied groups of mother rats and their offspring. Note: a highly significant in the levels of SOD, CAT and GPx (with the exception of GPx in offspring) and significant increase in the level of MDA among CP treated group. Following administration of CP and garlic extract, the levels of SOD, CAT, GPx and MDA are restored near to the control values. *(The software used to edit the images and labels: Adobe photoshopCS8me* similar results can be achieved using Microsoft Paint *).*

The level of SOD appeared significantly higher (p < 0.001) in the renal tissues of mothers that were supplemented with garlic extract alone while that of offspring appeared with non-significant change (p > 0.001) if compared with cotrol. In CP-treated mothers rats and their offspring, the level of SOD showed a remarkably significant decline (p < 0.001) if compared with the control and garlic groups. In CP-treated mother rats receiving garlic extract, the renal SOD significantly decreased if compared with CP-treated alone but still significantly lower than control; however, their offspring had with non-signifcant (p > 0.001) changes ( Table 2 & Figure 3A&A1).

**Table 2.** The levels of malondialdehyde (MDA), superoxide dismutase (SOD), catalase (CAT), and glutathione peroxidase (GPx) in the renal tissues among the different studied groups of mother's arts and their offspring

Parameter		Groups			
		Control n=6	Garlic n=6	Cisplatin (CP) n=6	CP & Garlic n=6
MDA (Nmol/mg tissue protein)	Mothers	0.47 ± 0.02	0.36 ± 0.028 ^a(^ ^**^ ^)^	0.63 ± 0.015 ^a(^ *** ^)^	0.49 ± 0.016 ^b(^ *** ^)^
	21 day-offsprings	0.49 ± 0.018	0.42 ± 0.017 ^a(^ ^*^ ^)^	0.70 ± 0.017 ^a(^ *** ^)^	0.53 ± 0.016 ^a(^ ^*^ ^),b(^ *** ^)^
SOD (U/mg tissue protein)	Mothers	9.13 ± 0.11	10.47 ± 0.13 ^a(^ ^**^ ^)^	6.11 ± 0.11 ^a(^ *** ^)^	8.095 ± 0.2 ^a(^ ^*^ ^),b(^ *** ^)^
	21 day-offsprings	17.89 ± 0. 16	18.49 ± 0. 15	12.15 ± 0. 17 ^a(^ *** ^)^	18.31 ± 0. 14
CAT (U/mg tissue protein)	Mothers	0.34 ± 0.11	0.42 ± 0.13 ^a(^ *** ^)^	0.218 ± 0.17 ^a(^ *** ^)^	0.39 ± 0.008 ^a(^ ^*^ ^),b(^ *** ^)^
	21 day-offsprings	0.077 ± 0. 008	0.15 ± 0. 009 ^a(^ *** ^)^	0.024 ± 0.006 ^a(^ *** ^)^	0.07 ± 0. 013
GPx (U/mg tissue protein)	Mothers	3.77 ± 0.11	4.08 ± 0.12	1.55 ± 0.057 ^a(^ *** ^)^	3.19 ± 0.096 ^a(^ ^*^ ^),b(^ *** ^)^
	21 day-offsprings	2.92 ± 0. 12	2.96 ± 0. 12	2.95 ± 0. 12	2.95 ± 0. 09

Data are expressed as means ± Standard error, (n=6 per group). Statistical analysis: one-way ANOVA followed by post hoc test. Means in the same row with different superscript letters are significantly different (p < 0.05). When p < 0.05,

^*^ Significant at p-value ≤ 0.05,

^**^ Significant at p-value ≤ 0.01 and

^***^ Significant at p-value ≤ 0.001.

In garlic extract treated mother rats and their offspring the level of renal tissues CAT appeared significantly higher than control (p < 0.001). However, a highly significant decline (p < 0.001) of this enzyme was noticed in the CP-injected mothers and their offspring. Concomitant administration of CP and garlic extract lessened such change in the CAT enzyme induced by CP, especially in offspring rather than mothers. Despite mothers still showing a significant decrease (p < 0.001) compared to the control ( Table 2 & Figure 3 B&B1).

In CP-treated mothers, the renal content of GPx appeared significantly lower than the control (P<0.001). Post-treatment of CP-treated mother rats with garlic extract, the level of GPx enzyme showed significant elevation if compared with CP-treated group but still with a low significance decrease if compared with the control. The offspring of all studied groups appeared with non-significant changes from the control ( Table 2 & Figure 3C&C1).

In garlic extract supplemented mother rats and their offsprings, the level of MDA in renal tissue homogenate appeared significantly lowered (p < 0.001) if compared with the control. In CP-treated mothers and their offspring the level of MDA showed highly a significant increase (p < 0.001) if compared with control. Following administration of CP and garlic extract, the level of MDA showed non-significant change (p > 0.001) compared with the control ( Table 2 & Figure 3 D&D1).

### The activity of caspase-3 in renal tissues.

**Figure 4.**
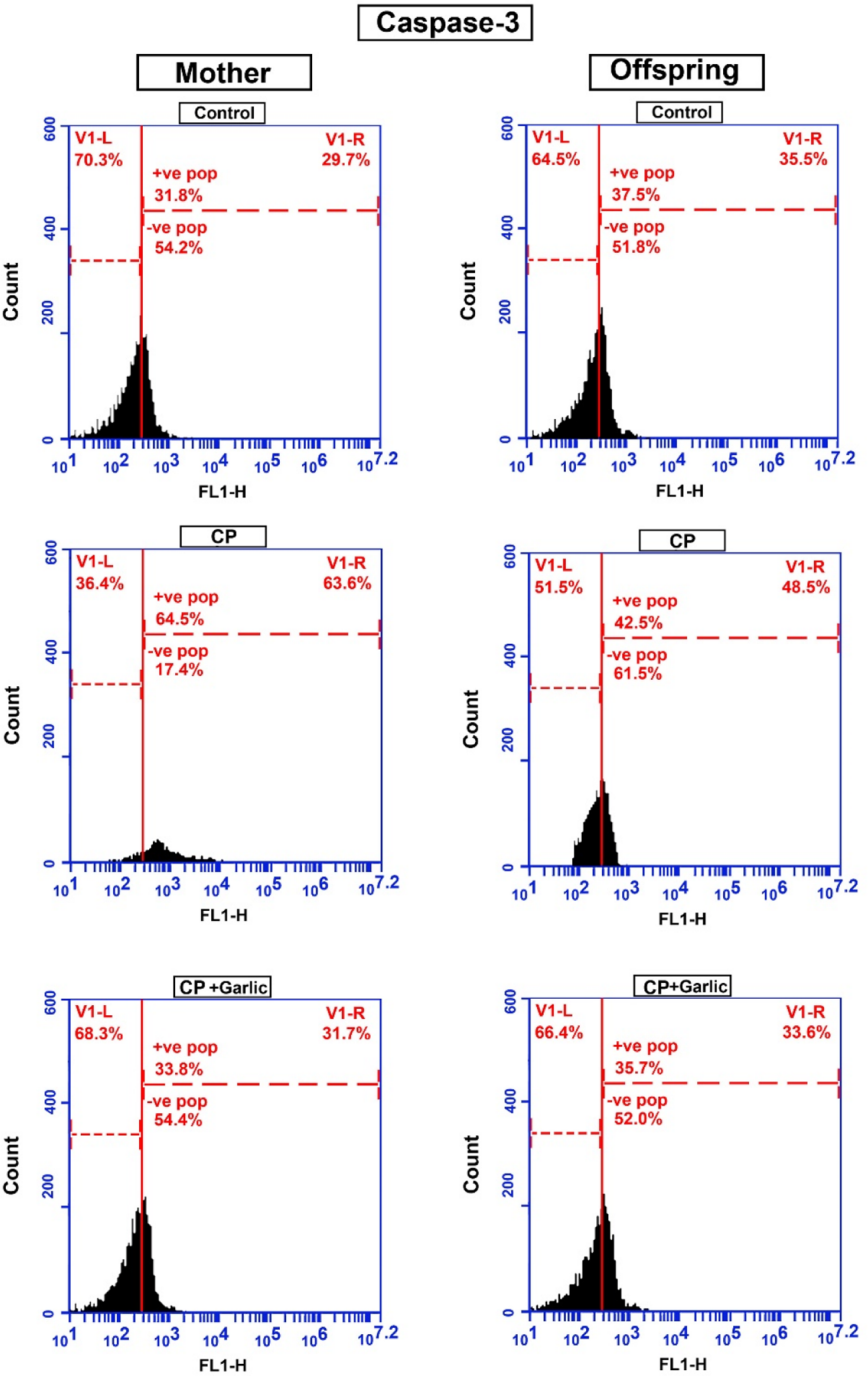
A flow cytometric chart showing the mean % value of caspase-3 activity in the renal tissues of mother rats and their offsprings. Note a highly % value of caspase3 activity in cisplatin (CP) induced mother rats and their offspring (63.6%, 48.5%) if compared with their control (29.7%, 35.5%) respectively. Following administration of CP and garlic extract, the mean % value of caspase-3 activity in the renal cells significantly lowered (mother rats =31.7%%, Offsprings =33.6%) if compared with CP-treated group. *(The software used to edit the images and labels: Adobe photoshopCS8me* similar results can be achieved using Microsoft Paint *).*

The flow cytometric data, in the current research, displayed that the normal percentage value of apoptosis caused by caspase-3 in the renal tissues of control mother was lower than that of their offsprings (29.7% and 35.5%) respectively. However, the mean percentage value of apoptosis induced by activated caspase-3 was increased in CP-treated mother rats and their offsprings (63.6% and 48.5% respectively) if compared with control. Following administration of CP and garlic extract markedly declined the elevated percentage value of apoptosis caused by activation of caspase-3 to (31.7% and 33.6% in mothers and offspring respectively) ( Table 3 & Figure 4).

**Table 3.** The mean % value of caspase-3 activity in the renal tissue of mothers' rats and their offspring.

N=6	Control	Cisplatin (CP)	CP+Garlic
Mother	29.7%	63.6%	31.7%
21-day offspring	35.5%	48.5%	33.6%

## Discussion

CP has severe deleterious side effects on pregnant mothers and their fetuses. ^40^^,^^41^ On the other hand, it had been emphasized that carrot flavor like garlic and several other flavors are transmitted from the lacataing mother to her fetuses. ^42^ Accordingly, the current work aimed to evaluate the modulatory role of garlic extract against CP-iduced nephrotoxicity in pregnant mother rats and their offsprings.

The obtained results of the current study revealed a remarkably significant decrease in the final body weight of CP-treated mother rats and their offspring if compared with a control. This result came in accordance with findings of previous studies ^43^^,^^44^ who recorded a significant decrease in the body weight of CP-treated rats treated with a single dose (7.5 mg/kg). The declined Bwt in CP-treated rats might be attributed to the direct damage of CP on renal tubular cells resulting in decreased water and sodium reabsorption with subsequent polyuria, dehydration. ^45^^,^^46^ Another study postulated that the decreased body weight in CP injected rats might be attributted to gastrointestinal toxicity resulting in lost appetite, ingestion and assimilation of food. ^48^ The reduction in body weight of offspring which their mothers received CP may be attributed to excretion of CP metabolites in milk during breast feeding. On the other hand, an obvious increase of body weight was noticed postsupplementation garlic extract to CP treated -mother rats. Nasr and Saleh reported that garlic extract can help maintain body weight through regulation of renal and intestinal enzyme functions. ^33^ Another study revealed that, if mothers consumed certain flavor compounds during the gestation and breastfeeding period, this would allow assimilation of carrot-flavored cereals to become available to their infants as infants are unable to digest this themselves. ^48^ A further study assumed that, ingestion of garlic by pregnant women leads to longer breast attachment for their infants. ^49^ In contrast to obtained result concerning body weight, Dixit and Joshi recorded a significant reduction in body weight after supplemattion with a garlic extract. ^50^ Such conflicting results may be attributed to the difference in exposed dose of garlic as well as the conditions of experiment.

Deleterious histological changes appeared in the renal cortex of CP-treated mother rats and their offspring. Such changes included atrophied glomeruli, dilatated Bowman’s space, necrosis and detachment of the proximal tubular cells, dilated tubular lumin of both proximal and distal tubules. This could be due to the cytotoxic effect of CP. Similar observations were recorded in rats treated with different doses of CP. ^51^^,^^52^ Aydogan *et al*. postulated that CP causes direct cellular damage on the renal corpuscles and tubules structures through generation of reactive oxygen species (ROS). ^53^ It had been reported that the nephrotoxicity induced by CP in childhood cancer therapy is accompanied with renal tubular damage, excess elimination of low molecular weight peptides and decreased excretion of some glycoprotein protein. ^54^ Another study on CP revealed that about 25% of patients have a significant increase in the levels of blood nitrogen following 1-2 weeks of treatment which is considered as a main cause for glomerular and tubular damage. ^55^ Pinta and Lippard suggested that the accumulation of platinum metabolites of CP inside the renal tubule is implicated in induction of nephrotoxicity. ^56^ Moreover, frequent doses of CP can directly induce oxidative stress renal tubular and glomerular cells resulting in inflammation and apoptosis. ^57^

In the present study, a remarkable improvement was recorded in the renal tissues of mother rats administrated with garlic after treatment with CP. Previous reports have demonstrated that garlic extract can prevent the oxidative stress and exerts amelioartive effect against various toxic agents through its vital antioxidant and free radical scavenger’s constituennts. ^58^^,^^59^ Other studies revealed that garlic has a powerful cytoprotective effects on the cells of vital body organs. ^60^^,^^61^

Serum creatinine and urea levels are considered the major biomarkers that efficiently mark the glomerular filtration rate. ^46^ In the current work, renal damage of CP was apparent from the increased levels of serum urea and creatinine in mother rats and their offspring. These observations agree with the previous, similar, reports. ^45^^,^^47^ Motegi *et al*. reported that, use of CP by pregnant women can exhibit a reversible elevation of serum creatinine and urea of her neonates. ^62^ Furthermore, the elevation in the serum levels of creatinine and urea might be attributed to renal disfunctions, ^58^ tubular obstruction and cytotoxicity of the renal tubules cells. ^46^ The disturbance in renal functions by CP is mainly due to its ability to supress protein synthesis by the renal tubular cells, ^63^ or to promote lipid peroxidation and liberation free radicals in renal tubular cells. ^64^ Aydogan *et al*. added that the significant elevation in the renal biomarkers is mainly attributed to the direct cytotoxic effect of CP on the glomerular and tubular structures through the excessive liberation of ROS. ^53^

In the present study, administration of CP followed by garlic extract revealed obvious recovery in the levels of serum creatinine and urea that markedly elevated by treatment with CP alone. This could be attributed to a cytoprotective effect of garlic. Our findings are parallel to previous reports. ^33^^,^^58^ Razo-Rodriguez *et al*. assured that garlic extract can maintain the levels of serum urea and creatinine through its organo-sulfur compounds. ^65^ Moreover, these compounds could enhance the antioxidant effect and inhibit the levels of lipid peroxide through scavenging the free radicals and elevation of intracellular concentration of glutathione.

The present study revealed a significant decrease in the serum sodium and potassium in CP-treated mother rats and their offspring. Such results came in agreement with the findings of previous studies ^66^^,^^67^ which showed excess elimination of potassium and sodium in patients treated with CP. An early study by Daugaard *et al*. confirmed that, CP metabolites can inhibit the mechanism involving electrolytes reabsorption especially in the distal segment of the nephron, resulting in hypokalemia and hyponatremia. ^68^ Furthermore, CP can induce disturbance in both intestinal absorption and renal tubular reabsorption of potassium. ^66^ Another study clarified that CPcan inhibit the activity of antidiurtic hormone leading to hypernatremia. ^69^

Garlic contains organo-sulfur compounds including S-Allylcysteine and allicin. These compounds had been reported to have a strong antioxidant role by elevation of GPx content in the cells as well as in scavenging of liberated free radicals. ^59^^,^^70^^,^^71^ Other related studies revealed that, garlic extract can alleviate the cardiotoxicity induced by doxorubicin, ^72^ Cd-induced toxicity ^52^ and oxidative stress induced by acrylamide in different body organs. ^73^ In the current work, treatment of mother rats with garlic extract alone or in combination with CP resulted in significant decline in the level of MDA and significant elevation of SOD, CAT and GPx activities in the kidney tissues. These findings support the antioxidant effects of garlic extract against CP-induced oxidative damage and lipid peroxidation. Lanzotti revealed that the thiosulfinates compounds of garlic play a major role in enhancing the antioxidants enzymes. ^74^ Other constituents of garlic like S-allyl mercaptocyteine, and selenium have been reported to have potent antioxidant activity. ^59^ Furthermore, S-allyl cysteine can inhibit the excessive liberation of lipid peroxidation and consequently stimulate potent antioxidant effects in both *in vitro* and *in vivo* experiments. ^75^ In agreement with present study, significant elevation of CAT, SOD and GPx activities accompanied by marked decline in MDA was recorded in animals treated with garlic. ^76^^,^^77^ The obtained results in the present work concerning the reduction in the activity of renal antioxidant enzymes; CAT, SOD and GPx and elevated lipid peroxidation (MDA) in CP treated mother rats are in line with the findings of previous reports. ^65^^,^^78^ Sheikh-Hamad *et al*. declared that CP can liberate nitric oxide that is implicated in oxidative stress and nephrotoxicity. ^79^ Kart *et al*. added that CP is implicated in excessive production of ROS in renal cell damage by promoting disturbance in membrane permeability. ^80^ Elevation of the MDA enhanced lipid peroxidation and increased ROS generation leading to disturbance of membrane function and integrity. ^81^ Moreover, the inhibitory effect of CP on CAT, SOD, and GPx was implicated in the pathogenesis renal tissues.

Caspases are a family of proteases that play an important role in the regulation of apoptosis. ^82^ In the current investigation, caspase-3 (strong apoptotic marker) has been shown to be increased in the renal tissues of CP-treated mother’s rats and their offspring. The obtained result agrees with work by previous researches. ^82^^,^^83^ Sheikh-Hamad *et al*. reported that, CP can induce apoptosis through activation of tumor necrosis factor or messages form caspases 1, 2, 3 and 8 that activate caspase-3 in kidney. ^79^ Razzaque *et al*. revealed that CP increased expression of both Fas and Fas ligand in human proximal tubular epithelial cells which accelearate tha activity of caspse-3. ^84^ Other researchers reported that, CP can induce apoptosis in the renal tubular cells resulting in activation of caspase-9, which is a good promoter for the mitochondrial apoptotic pathway. ^83^^,^^85^ Further studies explained that CP can induce apoptosis with progressive accumulation of DNA and inhibition of DNA repair pathways, through generation of reactive oxygen species. ^57^^,^^86^

Garlic was found to attenuate the increased caspase-3 levels induced by CP treatment. Previously, it was reported that, s-allyl cysteine in garlic can prevent the lipid peroxidation and the oxidative damage to DNA resulting in inhibition of apoptosis. ^33^^,^^87^

## Conclusions

In light of the demonstrated findings in this investigation, protective imprints of garlic extract against CP-induced deleterious histological and biochemical changes were found. Garlic extract has powerful ameliorative effects on CP-induced oxidative stress and renal damage through its antioxidant, anti-inflammatory and antiapoptotic properties through post-treatment with garlic extract. Eventually, further studies with different dose regimens and on larger number of animals over longer durations are recommended.

## Data availability

### Underlying data

Figshare: Raw data for body weight and biochemical parameters. https://doi.org/10.6084/m9.figshare.19435616.v2 ^88^

This project contains the following underlying data:

- Raw data of biochemical parameters.xlsx (Raw data on rats body weight)

Figshare: Data of Flow cytometry detection of caspase-3.xlsx https://doi.org/10.6084/m9.figshare.19435676.v4 ^89^

This project contains the following uunderlying data:

- Raw data of flow cytometric analysis for caspase-3.xlsx

Figshare: Underlying data.pdf. https://doi.org/10.6084/m9.figshare.19435370.v1 ^90^

This project contains the following underlying data:

- Underlying data.pdf (Images/Figures used in the manuscript)

## Reporting guidelines

Figshare: Author Checklist – Full.pdf https://doi.org/10.6084/m9.figshare.19446926.v1. ^91^ This project contains the following reporting guidelines:

- Author Checklist – Full.pdf (ARRIVE checklist)

Data are available under the terms of the Creative Commons Attribution 4.0 International license (CC-BY 4.0).

The underlying image data for this study are too large to share openly. The image files are predominately JPG files and approximately **198 MB** in size. Considering the large size and multiple images **(n=204)**, the image files will be shared on request to readers. Please contact the corresponding author beltagyaaa@yahoo.com, if you would like to request access to the image files. Representative images are shown in the figures and can be found in the Figshare repository.

## Consent to participate

Not applicable.
