# Supplementary material for: Modulatory role of garlic ( Allium sativum) extract against cisplatin- induced nephrotoxicity in female albino rats and their offspring
Source: F1000Res. 2022 May 9;11:504. [Version 1] doi: 10.12688/f1000research.111293.1 (PMC10403747; doi:10.12688/f1000research.111293.1)
Supplement: Peer review history

# Peer review: Reviewer response for version 1

**DOI:** 10.5256/f1000research.122996.r188353

**Published:** 2023-08-04

**Authors:** Joyce Trujillo

**Affiliations:** 1 National Council on Science and Technology (CONACyT)-Instituto Potosino de Investigación Científica y Tecnológica-División de Materiales Avanzados, San Luis, Mexico

The authors need to elaborate on how the decapitation method ensures minimal blood loss, followed by the successful execution of cardiac puncture without any significant blood volume loss.

 To enhance clarity, the authors should standardize the axis titles and the labels for panels D and D1 of Figure 1. This consistency should extend to the specific electrolyte under evaluation. Furthermore, the description of Figure 1 would benefit from including CP and garlic extract doses. The authors should explain the absence of hypomagnesemia in the mother’s rats and their offspring, as this is a crucial aspect of this model that warrants discussion.

 For a more streamlined presentation of data, consider consolidating weight, creatinine, and electrolyte results into a single format, such as a table or figures. Considering its representational value, I lean towards favoring figures. If both a table and a figure are to be retained, in the case of Figure 3 and Table 2, align the presentation of results in a consistent order.

 The authors need to analyze whether the observed beneficial effects of garlic stem come directly from its antioxidant, anti-apoptotic, or anti-inflammatory properties, as suggested, and not by the possibility that these effects result from lowered serum levels of cisplatin via chelation or other chemical reactions.

 Emphasize the significance of incorporating a marker for renal tubular damage, particularly given that the filtration of CP is in the proximal tubules, such as Kim-1 or NAG. This tubular damage could be quantified subjectively through serum creatinine and urea level determinations.

Is the work clearly and accurately presented and does it cite the current literature?

Yes

If applicable, is the statistical analysis and its interpretation appropriate?

Partly

Are all the source data underlying the results available to ensure full reproducibility?

Yes

Is the study design appropriate and is the work technically sound?

Partly

Are the conclusions drawn adequately supported by the results?

Partly

Are sufficient details of methods and analysis provided to allow replication by others?

Yes

Reviewer Expertise:

Renal pathophysiology and metabolic diseases

I confirm that I have read this submission and believe that I have an appropriate level of expertise to confirm that it is of an acceptable scientific standard, however I have significant reservations, as outlined above.

# Peer review: Reviewer response for version 1

**DOI:** 10.5256/f1000research.122996.r188352

**Published:** 2023-08-01

**Authors:** Stanila Stoeva

**Affiliations:** 1 Medical university of Varna, Bulgaria, Bulgaria

I find the work presented intriguing! On the one hand, cancers are discussed, which are one of the most common causes of mortality worldwide. On the other hand, the authors present the possibilities of conducting effective pharmacotherapy based on biologically active substances of natural origin. Anyway, I believe that answering the following questions will increase the quality of the scientific project and increase its publicity, respectively:

1. Please describe how and by whom the plant material used was botanically characterized.
2. Did you define the algorithm of the extraction procedure or did you use someone else's protocol? If it is part of your laboratory's operating procedures, please explain the choice of conditions. If you have used a foreign protocol, please cite it in the manuscript. Add in the text at what temperature the extraction was carried out.
3. How is the plant extract used standardized? As you know, this is a key argument in justifying the observed biological effects. Perhaps the following article will give you guidance on the standardization procedure: A HPLC-UV Method for Analysis of Total Plant Extract and Catechin Fraction of *Bancha* Green Tea ^1^?

Is the work clearly and accurately presented and does it cite the current literature?

Yes

If applicable, is the statistical analysis and its interpretation appropriate?

I cannot comment. A qualified statistician is required.

Are all the source data underlying the results available to ensure full reproducibility?

Partly

Is the study design appropriate and is the work technically sound?

Yes

Are the conclusions drawn adequately supported by the results?

Yes

Are sufficient details of methods and analysis provided to allow replication by others?

Partly

Reviewer Expertise:

Isolation, HPLC analysis and in vivo assessment of plant extracts

I confirm that I have read this submission and believe that I have an appropriate level of expertise to confirm that it is of an acceptable scientific standard, however I have significant reservations, as outlined above.

## References

[1] : A HPLC-UV Method for Analysis of Total Plant Extract and Catechin Fraction of Bancha Green Tea. *The Natural Products Journal* .2023;13(2) : 10.2174/2210315512666220512212448 10.2174/2210315512666220512212448

# Peer review: Reviewer response for version 1

**DOI:** 10.5256/f1000research.122996.r146986

**Published:** 2022-10-03

**Authors:** Fahmy G. Elsaid

**Affiliations:** 1 Zoology Department, Faculty of Science, Mansoura University, Mansoura, Egypt

Thank you for your invitation to undergo the review of the article entitled: Modulatory role of garlic ( *Allium sativum*) extract against cisplatin-induced nephrotoxicity in female albino rats and their offspring. The article aimed to evaluate the possible ameliorative role of garlic extract against nephrotoxicity induced by cisplatin (chemotherapeutic drug) in pregnant rats and the work is also extended to evaluate the complication on the offspring and attenuated the nephrotoxicity using a natural product. The authors focused on the investigation of renal histopathological and biochemical changes induced by cisplatin. The obtained results revealed that garlic extract successfully alleviated the deleterious renal histopathological and functional signs induced by cisplatin. The authors discussed that the sulfur and other phytochemical constituents of garlic extract play an essential role in the amelioration of renal structure and function.

 The paper is interesting and suitable for publication in F1000Research after consideration of the following minor corrections.

1. The Histological sections of the control kidney mother need more resolution (Figure 2).
2. The authors need to explain the source of cisplatin and its concentration.
3.  How do the authors detect the dose of garlic extract?

Accordingly, I recommended this article be suitable for indexing.

Is the work clearly and accurately presented and does it cite the current literature?

Yes

If applicable, is the statistical analysis and its interpretation appropriate?

Yes

Are all the source data underlying the results available to ensure full reproducibility?

Yes

Is the study design appropriate and is the work technically sound?

Yes

Are the conclusions drawn adequately supported by the results?

Yes

Are sufficient details of methods and analysis provided to allow replication by others?

Yes

Reviewer Expertise:

Molecular physiology

I confirm that I have read this submission and believe that I have an appropriate level of expertise to confirm that it is of an acceptable scientific standard.
